# A novel deep learning segmentation model for organoid-based drug screening

**DOI:** 10.3389/fphar.2022.1080273

**Published:** 2022-12-14

**Authors:** Xiaowen Wang, Chunyue Wu, Shudi Zhang, Pengfei Yu, Lu Li, Chunming Guo, Rui Li

**Affiliations:** ^1^ School of Information, Yunnan University, Kunming, China; ^2^ School of Life Science, Yunnan University, Kunming, China; ^3^ Department of Radiation Oncology, Sichuan Cancer Hospital and Institute, Sichuan Cancer Center, School of Medicine, University of Electronic Science and Technology of China, Chengdu, China

**Keywords:** bladder cancer organoid, organoid segmentation, deep learning, RDAU-Net model, drug screening

## Abstract

Organoids are self-organized three-dimensional *in vitro* cell cultures derived from stem cells. They can recapitulate organ development, tissue regeneration, and disease progression and, hence, have broad applications in drug discovery. However, the lack of effective graphic algorithms for organoid growth analysis has slowed the development of organoid-based drug screening. In this study, we take advantage of a bladder cancer organoid system and develop a deep learning model, the res-double dynamic conv attention U-Net (RDAU-Net) model, to improve the efficiency and accuracy of organoid-based drug screenings. In this RDAU-Net model, the dynamic convolution and attention modules are integrated. The feature-extracting capability of the encoder and the utilization of multi-scale information are substantially enhanced, and the semantic gap caused by skip connections has been filled, which substantially improved its anti-interference ability. A total of 200 images of bladder cancer organoids on culture days 1, 3, 5, and 7, with or without drug treatment, were employed for training and testing. Compared with the other variations of the U-Net model, the segmentation indicators, such as *Intersection over Union* and *dice similarity coefficient*, in the RDAU-Net model have been improved. In addition, this algorithm effectively prevented false identification and missing identification, while maintaining a smooth edge contour of segmentation results. In summary, we proposed a novel method based on a deep learning model which could significantly improve the efficiency and accuracy of high-throughput drug screening and evaluation using organoids.

## 1 Introduction

According to the statistics of the World Health Organization, there were 19.29 million new cancer cases worldwide in 2020, among which 4.57 million cases were in China, accounting for 23.7% of the total cases. At the same time, the number of deaths caused by cancer in China reached 3 million in 2020, accounting for 30% of the total number of cancer deaths worldwide (Ferlay et al., 2020). Bladder cancer is the sixth most prevalent cancer in the world, accounting for the greatest incidence of urogenital tumors in China. The large number of bladder cancer patients puts great pressure on the medical system in China as well as other medical facilities around the world. In response to noxious stimuli or injury of the urinary bladder, destruction of the urothelium architecture occurs which may cause cystitis and even bladder cancer (Liu et al., 2015; Qiao et al., 2014). The first-line treatment of bladder cancer still uses the primary generation drugs such as cisplatin, to increase DNA damage in rapidly dividing cells and, thus, destroy tumor cells (MULLENDERS et al., 2019), (SHIN et al., 2014), (KNOWLES and HURST, 2015). This treatment is simple, and many patients show a drug resistance phenomenon. Multidrug resistance is the result of the combined actions of multiple factors and pathways (Huang et al., 2020a). Therefore, screening for new anticancer drugs is critical, but the lack of experimental models limits the development of this research. With the development of modern medicine and advances in biological science and technology, we have ushered in the era of precision medicine. In 2009, the first mouse intestinal organoid model was established, opening a new chapter in organoid research (Barker et al., 2007), (Sato et al., 2009). Since then, organoid research has attracted considerable attention, and cell cultivation techniques have become increasingly sophisticated. Organoid research has been continually rated as one of the top 10 breakthroughs in scientific and technological development and one of the annual life science and technology by *Science* and *Nature Methods* (Foley, 2017).

Organoids are widely used to model key characteristics of organs and tissues to better understand various aspects of human disease, including cancer. The idea that organoids can model human pathologies has opened the door to studies on the feasibility of drug testing and screening applications (Rossi et al., 2018). As they have the characteristics of cell proliferation, self-renewal, and genetic stability, organoids can already mimic organs such as the brain, retina, and gut. Tumor organoids are directly derived from patients. These tumor cells are cultured *in vitro* and can simulate the tumor microenvironment *in vivo*, improving the accuracy of tumor drug screening (Ballard et al., 2019), (Clevers, 2016), (Souza, 2018), in which organoid construction and assessment of drug effects have been demonstrated. For example, Suk Hyung Lee’s team tested first-line therapeutic drugs on bladder cancer patient-derived organoids and found that the results were consistent with clinical presentation and that the drug response of the organoids could be validated in xenografts (Lee et al., 2018). Organoid technology has developed rapidly in the past decade. Through continuous optimization of culture methods, researchers have successfully cultivated a variety of tumor organoids, such as colorectal cancer (Cave et al., 2021), prostate cancer (Karkampouna et al., 2021), pancreatic cancer (Huang et al., 2020b), breast cancer (Dekkers et al., 2021), gastric cancer (Seidlitz et al., 2019), bladder cancer (Yu et al., 2021), and biliary carcinoma (Yuan et al., 2022).

In recent years, with the continuous development of artificial intelligence, deep convolutional neural networks represented by SegNet (Badrinarayanan et al., 2017), VGG (Simonyan and Zisserman, 2014), GAN (Goodfellow et al., 2014), and ResNet (He et al., 2016) are widely used in the field of computer vision. With the help of the convolutional network, significant progress has been made in the detection and classification of medical images, such as the detection and classification of fundus retinopathy (Pratt et al., 2016), the detection of tumor targets (BejnordiEhteshami et al., 2017), and the classification of lung nodules (Kumar et al., 2015), as well as other applications. This study aims to help drug screening by automatically identifying and segmenting organoids in images, calculating their area, and observing and recording their growth status. The current image segmentation methods are divided into two categories: traditional methods and deep learning-based segmentation methods. Traditional methods include threshold-based segmentation, watershed segmentation, and morphological operations (Jung and Kim, 2010). The advantage of the traditional method is that it does not require experts to manually annotate images and segmentation can be achieved quickly. However, it is difficult to find a suitable and reliable threshold in a complex background, which significantly reduces the segmentation accuracy of the algorithm, and thus such methods have significant limitations.

With the rapid development of deep learning techniques, various deep learning techniques based on segmentation algorithms are being applied to medical image segmentation. Shelhamer et al. (Long et al., 2015) used fully convolution networks (FCNs) in 2015 to perform pixel-level, end-to-end image segmentation tasks. FCNs can be regarded as the first work of network models in image segmentation. Subsequently, Ronneberger et al. (2015) proposed U-Net based on the FCN. The skip connection of this model can adequately compensate for the information loss problem in the down-sampling process of the FCN. Due to its simple structure, few parameters, and strong plasticity, U-Net is widely used in various image segmentation tasks, especially for medical image segmentation tasks with few samples. However, the segmentation effect of some detailed parts in medical images is still lacking. For example, in the retinal blood vessel segmentation task, the segmentation of small blood vessels at their termini may be broken or missing (Wang et al., 2022). Some researchers have improved upon the basic framework of U-Net. Quan et al. (2021) combined the U-Net with the residual structure (He et al., 2016), while improving the skip connection to construct a deeper model for segmentation. Some researchers have also gradually added attention mechanisms (Mnih et al., 2014), recurrent neural networks, and transformer structures (Vaswani et al., 2017) to segmentation networks. Attention U-Net (Oktay et al., 2018) adds a gate control at the skip connection, aiming to solve the semantic gap at feature splicing. R2U-Net (Nasrin et al., 2019) replaces the convolution module in U-Net with a convolution module with loop and residuals and enhances the performance of the model using feature accumulation. The transformer model was originally used in the field of natural language processing (NLP). Because of its excellent long-distance modeling capabilities to overcome the limitations of the convolutional network, some researchers now use the transformer model structure for computer vision. For instance, Chen et al. (2021) combined the transformer model with U-Net to construct TransUNet and achieved excellent segmentation results for the multi-organ segmentation dataset Synapse (which included eight abdominal organs: aorta, gallbladder, spleen, and kidney *.*).

**FIGURE 1:**
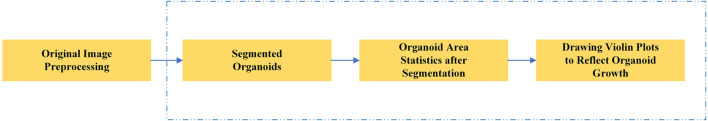
Main steps of the automated drug screening assessment in organoids. The steps in the blue dotted line show the traditional, manual method, while in our method these steps are performed automatically by the computer.

In this study, we proposed a novel deep learning segmentation model. Our model is based on the U-Net framework, which has improved feature extraction and recovery capability. After the segmentation of the organoids in the input image, the area of all organoids in the image is calculated to reflect the growth rate of organoids and evaluate the effect of related anticancer drugs. Figure 1 shows the main research steps. In the conventional method, the whole process is performed manually. In our method, the main steps can be performed automatically by the computer (steps in blue dotted line), which considerably improves the efficiency of drug screening.

## 2 Materials and methods

### 2.1 Datasets and data preprocessing

**FIGURE 2:**
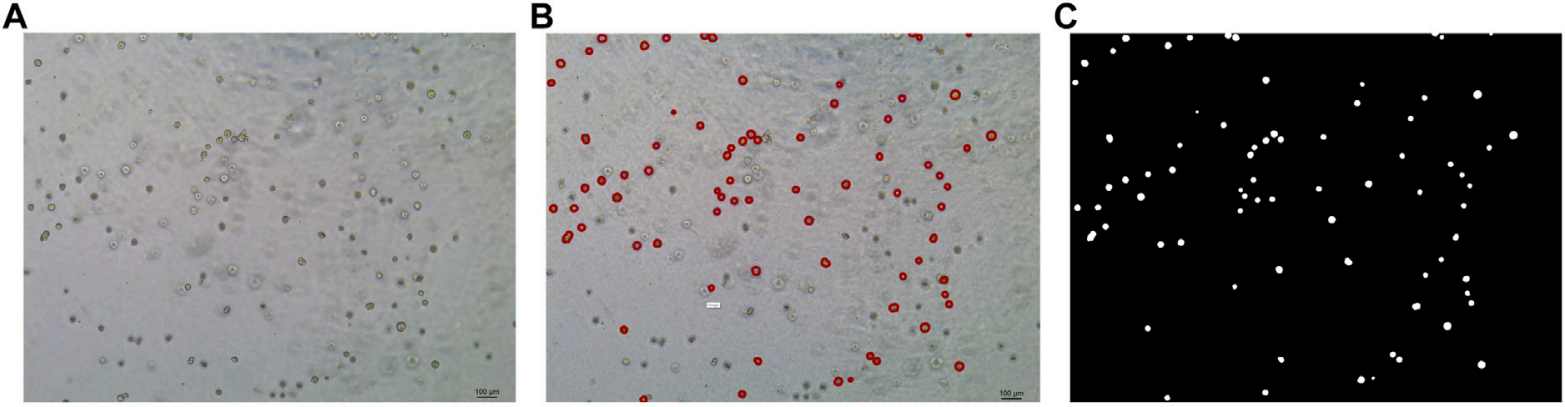
Steps for making labels. **(A)** Original image of organoids on the third day. **(B)** Manual labeling of organoids. **(C)** Label generation by LabelMe software for model learning. The white regions are the target organoids, and the black region is the background.

**FIGURE 3:**
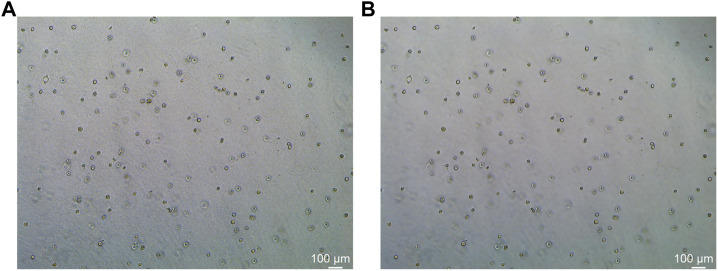
Comparison before and after non-local mean filtering. **(A)** Original image of organoids. **(B)** Image of organoids after preprocessing using the non-local mean filter. It can be observed that the background of the processed image is smoother.

In this study, the dataset used for analysis consists of organoid images of bladder cancer cell lines treated with different drugs for 1–7 days. We used the human bladder cancer cell line SW780, a cell line established by A. Leibovitz in 1974 from a first-stage transitional cell tumor, for 3D culture. When the cells were passaged, approximately 2000 cells were placed in Matrigel (R&D Systems) in a 24-well plate. The Matrigel drops were solidified for 10 min at 37°C and 5% CO_2_. Upon Matrigel solidification, 600uL of the SW780 medium was added to each well. The medium is the RPMI 1640 medium supplemented with 1% penicillin/streptomycin, 1% GlutaMAX, 1% HEPES, and 10% FBS. The drugs used, RA and 14, two derivatives of vitamin A, were diluted in the medium at a specific concentration, and the medium was replaced every 2–3 days. Images of organoids were captured by a Leica microscope at ×5 magnification. The images of bladder organoids collected in our study are characterized by uneven illumination, blurred boundary contours of some organoids, mutual adhesion of multiple organoids, and high background complexity. The 5X images were used for area statistics and analysis, which is also the dataset used in this study. The dataset at this stage contained 200 images, with sizes of 1944 × 2,592 pixels and 768 × 1,024 pixels. Deep learning-based image segmentation requires experts to manually draw labels for the model to learn. In this study, labels are created using the LabelMe software. First, the organoids to be segmented are marked and regenerated into a black and white binary image, as shown in Figure 2. Figure 2A shows the image of organoids on the third day. It can be observed that there are a large number of organoid ghosts and bubbles in the figure, which greatly increases the difficulty of model segmentation. The red-marked regions in Figure 2B are the organoids that have been manually identified. Figure 2C shows the final label for the model to learn. We preprocessed the images using a non-local mean filter (Buades et al., 2005) method to enhance the contrast between the organoids and the background, as well as reduce the interference of organoid ghosting, as shown in Figure 3. The filter uses the entire image for denoising by searching for similar regions in the image in terms of image blocks and then averages these regions to better filter out the Gaussian noise in the image.

### 2.2 Methods

#### 2.2.1 Model structure

**FIGURE 4:**
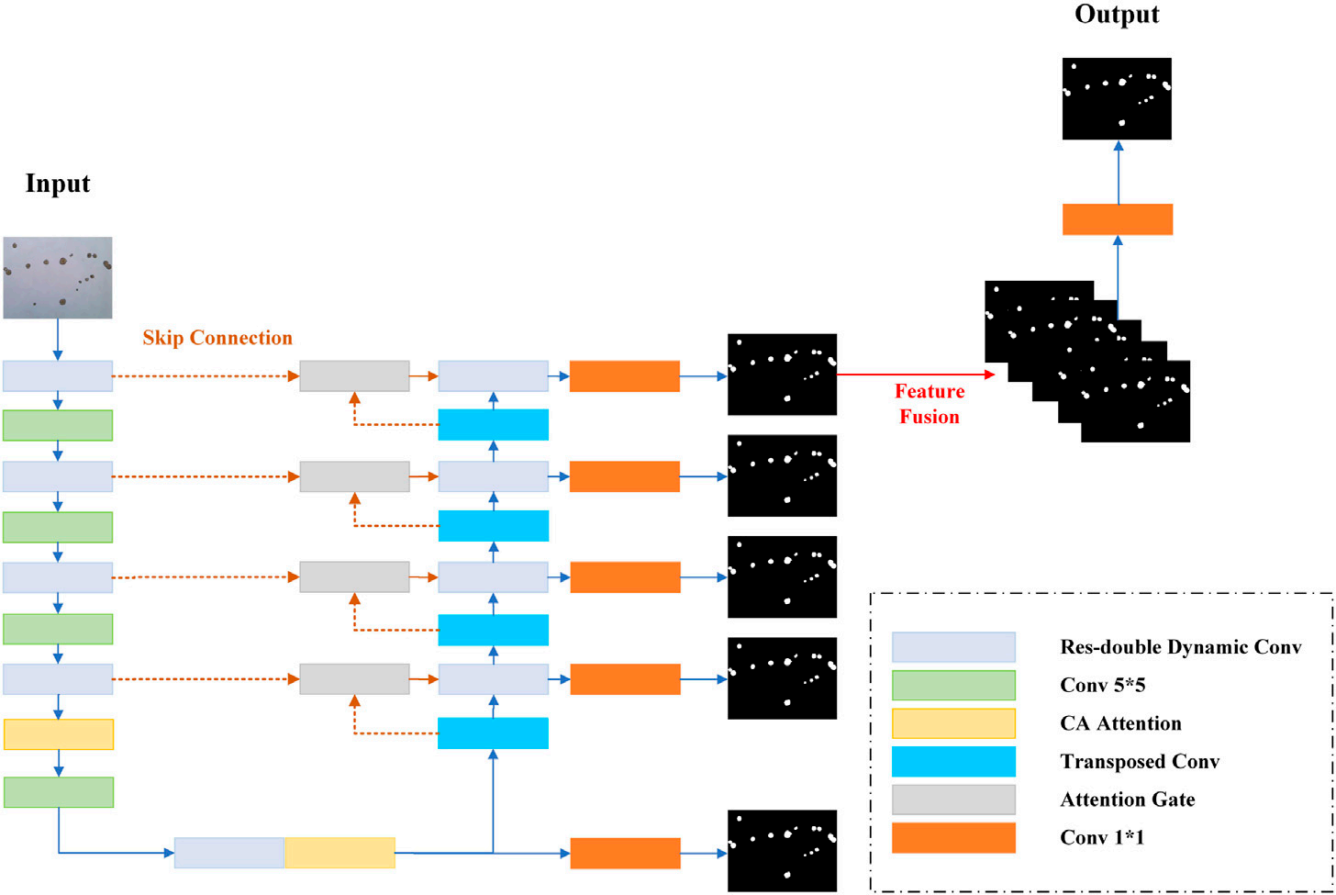
Schematic of the RADU-Net model structure.

In this study, we propose a res-double dynamic conv attention U-Net (RDAU-Net) model, using U-Net as the basic framework. The U-Net model structure is similar to sequence to sequence (Sutskever and Oriol VinyalsQuocLe, 2014), which is mainly composed of an encoder, decoder, and skip connections. The encoder performs feature extraction on the input image, which consists alternately of double-layer convolution and down-sampling. Accordingly, the decoder gradually recovers the extracted features to the same size as the original input image through up-sampling, which alternately consists of a double-layer convolution and up-sampling. The intermediate skip connections fuse the low-level features of the encoder part with the high-level features of the decoder part to reduce the information loss in the down-sampling process to obtain higher segmentation accuracy. Considering that most of the images in this dataset are of 1944 × 2,592 pixels, the receptive field of the double-layer convolution module of the original U-Net network needs to be enlarged. For this purpose, this study combines the residual structure and dilation rate dynamic convolution module (Chen et al., 2020) to replace the original double-layer convolution module and to increase the receptive field of the convolution module and the feature extraction ability of the encoder. In addition, the original max pooling down-sampling is replaced by a convolution with a convolution kernel size of five and a stride of 2, to further reduce the information loss in the down-sampling part of the encoder. Coordinate attention (CA) (Hou et al., 2021) is added after the dynamic convolution module of the last two layers of the encoder to refine and strengthen the extracted features. The skip connection part uses the attention gates in attention U-Net (Oktay et al., 2018) to weigh the features, suppress irrelevant regional features, and reduce the semantic gap. The decoder first fuses multi-scale feature information and convolves the output of each decoder layer, and then a concatenation operation is performed to adequately utilize the information at different scales in each layer of the decoder. It can combine high-level and low-level features for better feature recovery. Figure 4 shows the structure of the improved model. Each module is described in detail in the following sections.

#### 2.2.2 Res-double dynamic convolution

**FIGURE 5:**
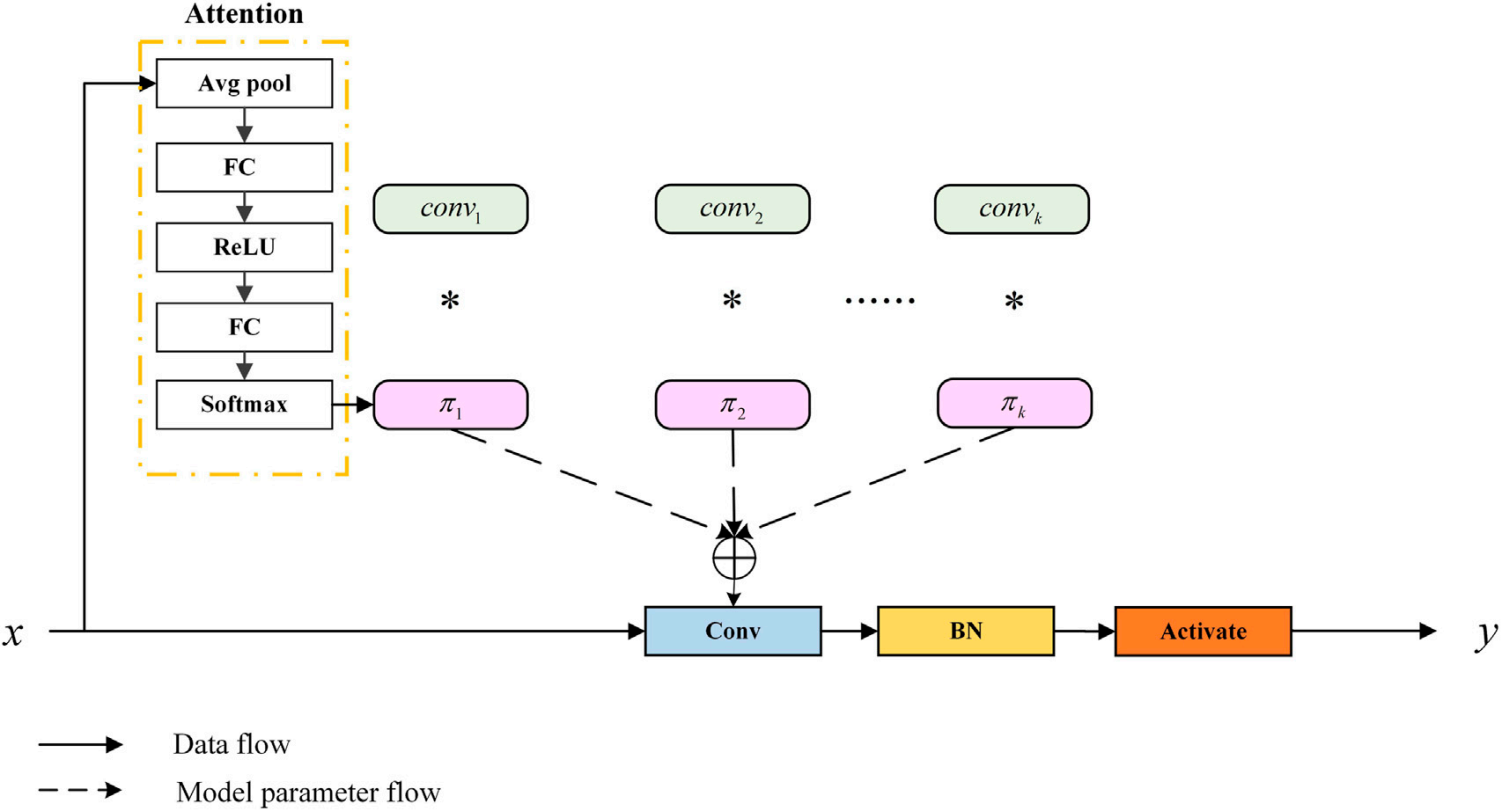
Schematic of the dynamic convolution module (Chen et al., 2020).

To allow the model to accurately capture the feature information of organoids, we applied dynamic convolution (Dy Conv). The classic standard convolution can also be called static convolution, whose parameters are unchanged and shared. In convolutional networks, the attention module generally acts on the feature map, weighing the channel and spatial position of the feature map. Dy Conv aggregates multiple parallel convolution kernels dynamically based on their attentions, which are input dependent. In other words, Dy Conv weighs the convolution kernel, and different convolution kernels can be used for different inputs. The purpose of Dy Conv is to find a balance between model performance and computational complexity. Usually, the way to improve the performance of convolutional networks is to widen and deepen, which consumes more computation. The Dy Conv module uses a more economical way to enhance network performance, and its structure is shown in Figure 5. The attention module in the structure is highly similar to squeeze-and-excitation networks (SENet) (Hu et al., 2018). SENet is to add channel attention, while Dy Conv is to add attention to the convolution kernel. The standard convolution is denoted as follows:

$$y=g\left(W^{T}x+b\right),$$ (1)

where W and b are the weight matrix and the bias vector, respectively, and g is the activation function. The Dy Conv aggregates multiple functions to define, as shown in the following equation:

$$\begin{array}{c} y=g\left({\tilde{W}}^{T}\left(x\right)x+\tilde{b}\left(x\right)\right)\\ \tilde{W}\left(x\right)=\overset{K}{\underset{k=1}{\sum }}\pi_{k}\left(x\right){\tilde{W}}_{k},\tilde{b}\left(x\right)=\overset{K}{\underset{k=1}{\sum }}\pi_{k}\left(x\right){\tilde{b}}_{k},\\ s.t.0\leq \pi_{k}\left(x\right)\leq 1,\sum_{k=1}^{K}\pi_{k}\left(x\right)=1 \end{array}$$ (2)

where $\pi_{k}$ is the attention weight of the ***k*-th** function ${\tilde{W}}^{T}x+\tilde{b}\left(x\right)$ , and $\pi_{k}$ varies with each input x . $\tilde{W}\left(x\right)$ and $\tilde{b}\left(x\right)$ are the weight matrix and bias vectors, respectively, which are formed by aggregating k parallel convolution kernels by attention weighting. They represent the best aggregation of linear models for a given input, and as the aggregated model is a non-linear function, Dy Conv has more feature expression capabilities than standard convolution.

**FIGURE 6:**
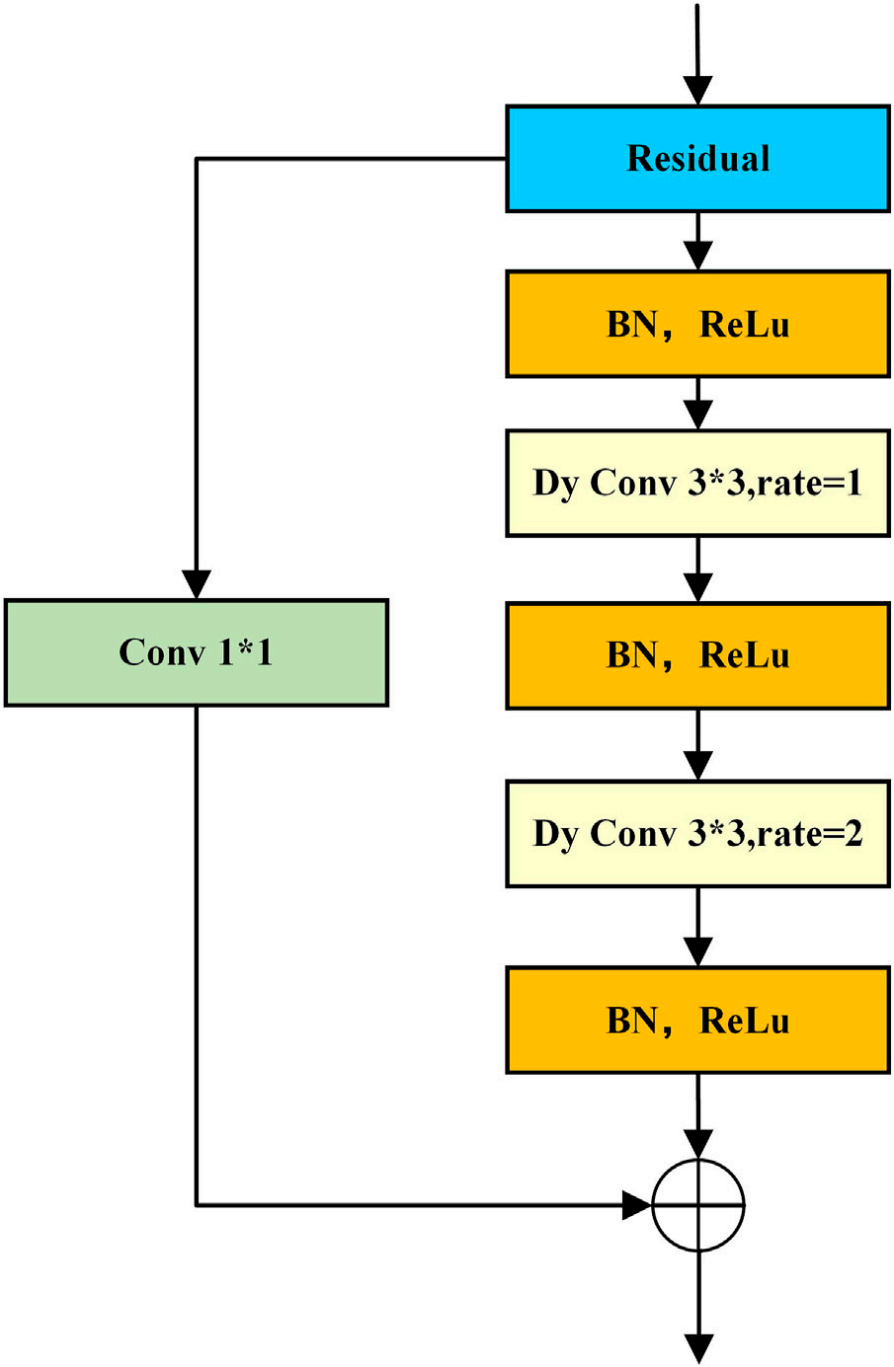
Schematic of the res-double dynamic convolution module.

To accurately segment organoids, the model should have a strong ability to learn global features and focus on target regions in complex backgrounds. Therefore, we applied the double-layer convolution module, which is composed of alternating three batch normalization (BN), three rectified linear unit (ReLU) activation functions, and two dynamic convolutions. The double-layer convolution is a res-double Dy Conv module structure, as shown in Figure 6. Although it adds a small amount of computational overhead, it can effectively enhance the feature extraction ability of the model. At the same time, the dilation rate is adopted to the dynamic convolution. This operation can further expand the convolution receptive field of the convolution without increasing the computational complexity. Combining convolutions with different dilation rates can also improve the model’s ability to learn multi-scale information. After experiments, the double-layer convolution module with a combination of dilation rates {1, 2} works best on this dataset. In addition, the residual structure is introduced to avoid performance degradation due to a large number of model layers, which makes the model more stable and accelerates convergence.

#### 2.2.3 Coordinate attention

The attention mechanism plays a crucial role in deep learning, which enables the model to suppress irrelevant region features and reinforce the target region features. As a result, we apply the attention mechanism in this study. The attention mechanism can improve the model’s ability to recognize target organoids and background artifact organoids. Two kinds of attention are used here: coordinate attention (CA) at the bottom of the model encoder and attention gate (AG) at the skip connections.

**FIGURE 7:**
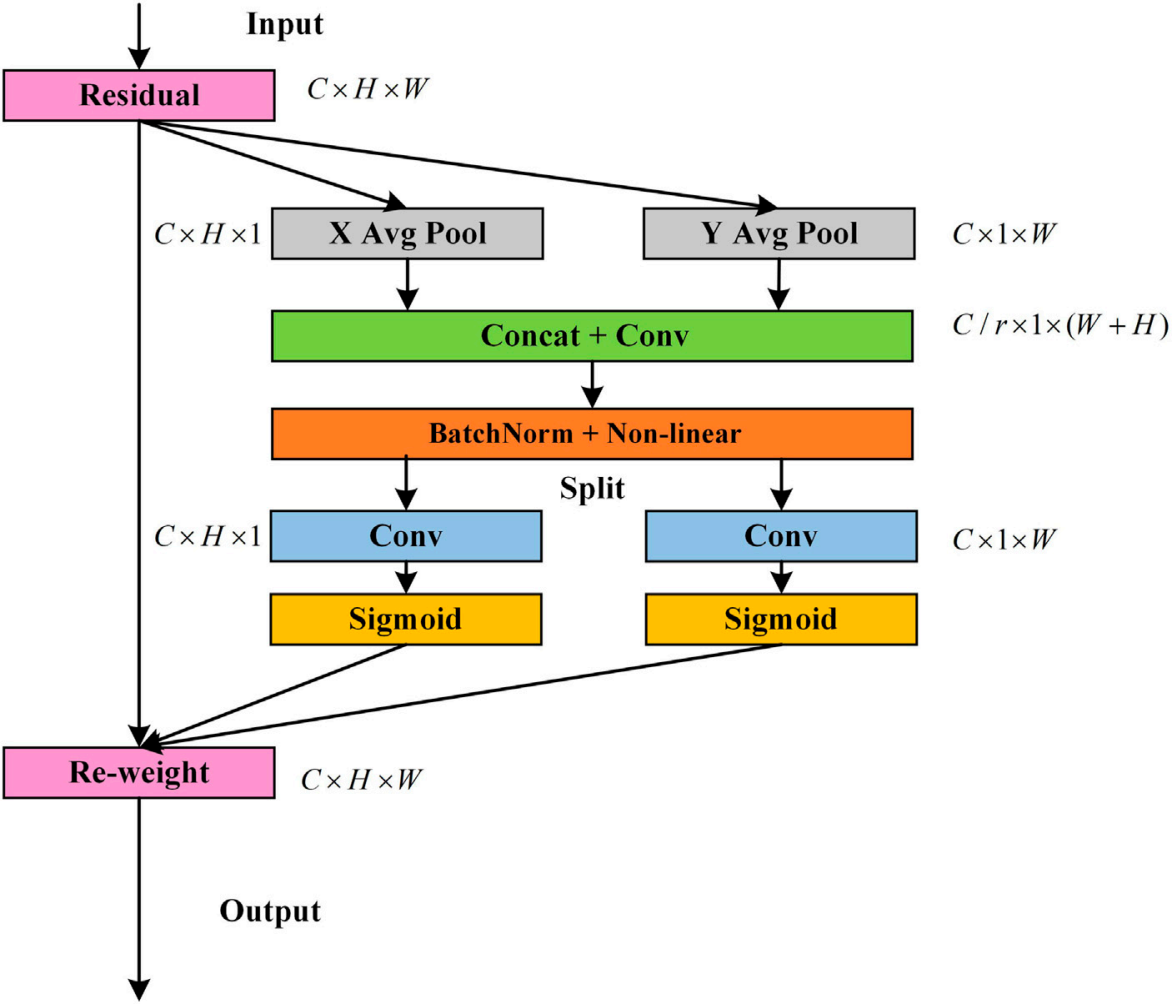
Schematic of the CA module (Hou et al., 2021).

The convolutional network is limited by convolution. It is difficult to capture long-range dependencies and global information cannot be effectively utilized, so the CA module is introduced to solve these issues. First, global average pooling is performed on both the height and width directions of the input feature maps to obtain one-dimensional feature maps in both directions, which enables the attention module to capture long-range spatial interactions with precise location information. The structure of the CA module is shown in Figure 7.

Assuming that the input size of the current feature map is C×H×W , global average pooling is performed on the height and width directions of the feature map, respectively. Thus, the output $z_{c}^{h}\left(h\right)$ of the ***c-*th** channel at height h and output $z_{c}^{w}\left(w\right)$ of the ***c-*th** channel at width w are written as follows:

$$z_{c}^{h}\left(h\right)=\frac{1}{W}\underset{0\leq i\leq W}{\sum }x_{c}\left(h,i\right),$$ (3)

$$z_{c}^{w}\left(w\right)=\frac{1}{H}\underset{0\leq j\leq H}{\sum }x_{c}\left(j,w\right).$$ (4)

Subsequently, they are concatenated in the spatial dimension, and then the convolution is used to reduce the number of channels and the activation function is applied to perform non-linear mapping, as shown in the following equation:

$$f=\delta \left(F_{1}\left(\left[z_{c}^{h},z_{c}^{w}\right]\right)\right),$$ (5)

where **[** $z_{c}^{h},z_{c}^{w}$ **]** denotes the concatenation operation along the spatial dimension, $F_{1}$ represents the convolution operation, and δ is the non-linear mapping of the ReLU function. f is then decomposed into two separate tensors $f^{h}$ and $f^{w}$ along the spatial dimension in the height and width directions. The two are restored to have the same number of channels as the original input feature map using 1 × 1 convolution transformations $F_{h}$ and $F_{w}$ . After the weight coefficient is compressed by the sigmoid function σ , it is finally multiplied by the original input image, as shown in the following equations:

$$g_{c}^{h}=\sigma \left(F_{h}\left(f^{h}\right)\right),$$ (6)

$$g_{c}^{w}=\sigma \left(F_{w}\left(f^{w}\right)\right),$$ (7)

$$y_{c}\left(i,j\right)=x_{c}\left(i,j\right)\times g_{c}^{h}\times g_{c}^{w},$$ (8)

where $g_{c}^{h}$ and $g_{c}^{w}$ are the attention weights in the height dimension and width dimension, respectively. $y_{c}\left(i,j\right)$ is output of the attention module. The CA module captures not only cross-channel information, but also features map spatial location information.

#### 2.2.4 Attention gate

**FIGURE 8:**
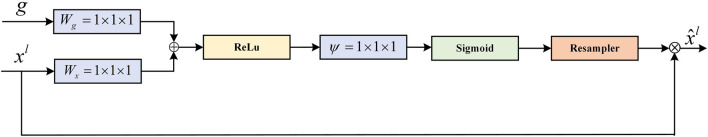
Schematic of the AG module (Oktay et al., 2018).

The AG (attention gate) module is derived from the attention U-Net to solve the semantic gap between high-level features and low-level features at the skip connection. Specifically, a gating signal is added to the encoder and decoder feature maps as a way to control the importance of features at different spatial locations in the feature maps. The AG also filters out and suppresses feature responses in irrelevant regions. The module structure is shown in Figure 8.

**FIGURE 9:**
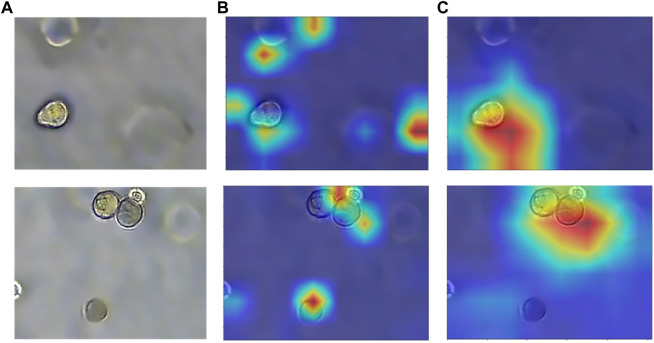
Heatmap with or without attention module. **(A)** Original image of organoids. **(B)** Heatmap without attention. **(C)** Heatmap with attention. The model with added attention is able to better focus on the target region.

The feature maps of the decoder and the feature maps of the previous layers of the encoder are used as the input to the module. Since the inputs come from different layers of the model, the required size and number of channels of the feature maps are adjusted to be the same. The element-wise addition is performed on two feature maps. This process can enhance the same regions of interest for input $x_{l}$ and g . Then the same regions of interest are enhanced by the ReLU activation function, and irrelevant regions are suppressed. Finally, the weight coefficient is calculated by the sigmoid function, and resampling restores the weight coefficient matrix to the same size as the input $x_{l}$ and then multiplies it to get $\hat{x_{l}}$ . The AG module adopts the idea of soft-attention, where the attention weight coefficient can be continuously adjusted as the model is trained. As shown in Figure 9B, the model without attention not only has a poor ability to capture the target regions but also is easily disturbed by the ghosting of organoids in the image. Also, Figure 9C shows that after adding the CA module and the AG module, the model can better focus on the target organoid regions, and the anti-interference ability is enhanced to achieve the effect of accurate segmentation.

#### 2.2.5 Experimental setting

In this study, 200 images with labels drawn by experts are divided into three sets: training set, validation set, and test set with a ratio of 14:3:3. Adaptive moment estimation (Adam) is used for training, and the loss function is weighted binary cross-entropy, the initial learning rate is 0.001, and the maximum number of iterations is 80. Considering that most of the pixels in an image of organoids to be segmented are background pixels, the pixels in the target region only account for an extremely small number. In this case of unbalanced pixel distribution, it will make the model heavily biased toward the background using the general cross-entropy function, resulting in a decrease in accuracy. The weighted cross-entropy function adds a weight parameter to each category based on the cross-entropy function to weight the positive samples, as shown in Equation 9:

$$WBCE\left(p,\hat{p}\right)=-\left(\beta p\log\left(\hat{p}\right.\right)+\left(1-p\right)\log(1-\hat{p})),$$ (9)

where β is the weight parameter which is adjusted to be less than one to reduce the number of false positives, p is the actual value, and $\hat{p}$ is the predicted value. All experiments are based on the PyTorch framework of Python3 to build the RADU-Net model. The experimental environment is the Ubuntu20.04 operating system, with an I9 7900 CPU and an NVIDIA RTX 3090 GPU with 24 GB of video memory.

#### 2.2.6 Evaluation indicators

The research applies five evaluation indicators to assess the performance of the model, including *accuracy* (*Acc*), *precision*, *recall*, *Intersection over Union* (*IoU*), and *dice similarity coefficient* (*DSC*). The *Acc* represents the proportion of correctly classified pixels to the total pixels. The *precision* represents the proportion of correctly classified organoid pixels to the total predicted organoid pixels. The *recall* represents the proportion of correctly classified organoid pixels to the actual total organoid pixels. The *IoU* and *DSC* measure the similarity between the segmentation result and the label. These indicators' calculations are shown as follows, and the meanings of the symbols in the formula (10) are shown in Table 1.

$$\begin{array}{c} \mathrm{Accuracy}=\frac{T_{N}+T_{P}}{T_{N}+T_{P}+F_{N}+F_{P}},\\ \mathrm{Precision}=\frac{T_{P}}{T_{P}+F_{P}},\\ \mathrm{Recall}=\frac{T_{P}}{T_{P}+F_{N}},\\ IoU=\frac{T_{P}}{T_{P}+F_{P}+F_{N}},\\ DSC=\frac{2T_{P}}{{2T}_{P}+F_{P}+F_{N}}. \end{array}$$ (10)

**TABLE 1:** Meaning of symbols in formula (10).

Symbol	Meaning
$T_{p}$	Number of correctly classified organoid pixels
$T_{N}$	Number of correctly classified background pixels
$F_{P}$	Number of background pixels misclassified as organoids
$F_{N}$	Number of organoid pixels misclassified as background

## 3 Results

In this section, the experiments are carried out to verify the superiority of the proposed RDAU-Net method. The data used in the following models’ segmentation performance comparison experiments are from the test set. To reduce random errors, all experiments in this study were repeated five times.

### 3.1 The RDAU-Net model

**FIGURE 10:**
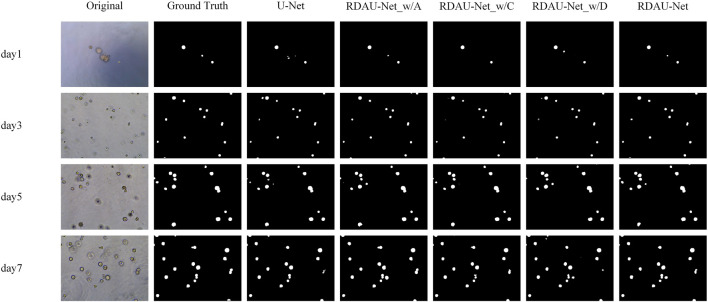
Segmentation results of different models for 1-, 3-, 5-, and 7-day images of organoids.

**FIGURE 11:**
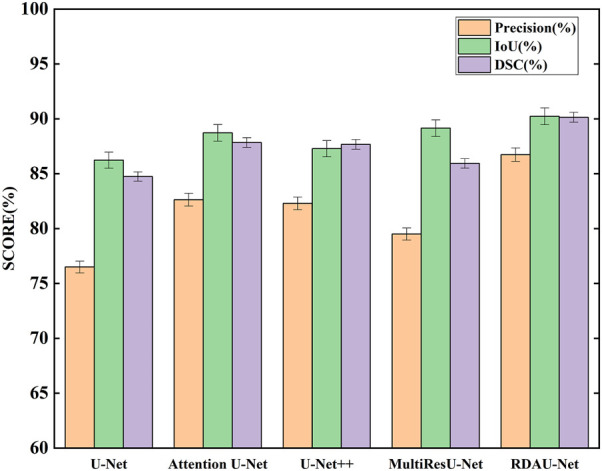
Comparison of segmentation indicators (*precision*, *IoU*, and *DSC*) of five models. Among the five models, RDAU-Net can achieve the best segmentation indicators.

The segmentation results of the models are shown in Table 2 and Figure 10. Here, the segmentation indicators of the eight models are compared, namely, U-Net, attention U-Net, U-Net++ (Li et al., 2020), MultiResU-Net (Ibtehaz and Sohel Rahman, 2020), RDAU-Net model, and its three ablation models. The segmentation accuracy of the RDAU-Net model on the test set can reach *Acc* of 99.32%, *recall* of 97.65%, *precision* of 86.73%, *IoU* of 90.23%, and *DSC* of 90.14%. The *IoU* of our model is 4.00%, 1.51%, 1.08%, and 2.95% higher than U-Net, attention U-Net, U-Net++, and MultiResU-Net, respectively. The *DSC* of our model is 5.40%, 2.30%, 2.47%, and 4.20% higher than U-Net, attention U-Net, U-Net++, and MultiResU-Net. On precision, it outperforms U-Net, attention U-Net, U-Net++, and MultiResU-Net by 10.23%, 4.10%, 4.43%, and 7.23%, respectively. These comparisons of the aforementioned indicators are shown in Figure 11.

**TABLE 2:** Segmentation indicators for eight models on the test set.

`	*Acc* (%)	*Precision* (%)	*Recall* (%)	*DSC* (%)	*IoU* (%)
U-Net	99.98	76.50	95.68	84.74	86.23
Attention U-Net	99.14	82.63	93.75	87.84	88.72
MultiResU-Net	99.02	79.50	94.73	85.94	87.28
U-Net++	99.11	82.30	93.55	87.67	89.15
RDAU-Net_w/A	99.27	83.03	97.09	89.51	90.13
RDAU-Net_w/C	99.26	83.53	96.31	89.47	90.09
RDAU-Net_w/D	99.17	79.97	97.27	87.78	88.68
**RDAU-Net**	**99.32**	**86.73**	**97.65**	**90.14**	**90.23**

The bold black is to highlight the contrast between our model and other models.

These indicators show that the improved RDAU-Net model has better segmentation accuracy. Reducing false identification when the image has a complex background is one of the difficulties in this study. Since the two indicators of *recall* and *precision* have a restrictive relationship, the addition of drugs was used to observe the growth status of organoids to reflect whether the added drug inhibits growth. False identification is a large error in the area of a calculation of organoids that cannot be truly and objectively reflected in organoid growth status. Therefore, this study focused on reducing the occurrence of false identification, that is, improving *precision* as much as possible under the condition of ensuring *recall*. It can be seen from the *precision* indicator that our model is increased by 10.23% compared with the U-Net basic model, which indicates that the phenomenon of model false identification is well controlled.

### 3.2 Ablation experiments

**FIGURE 12:**
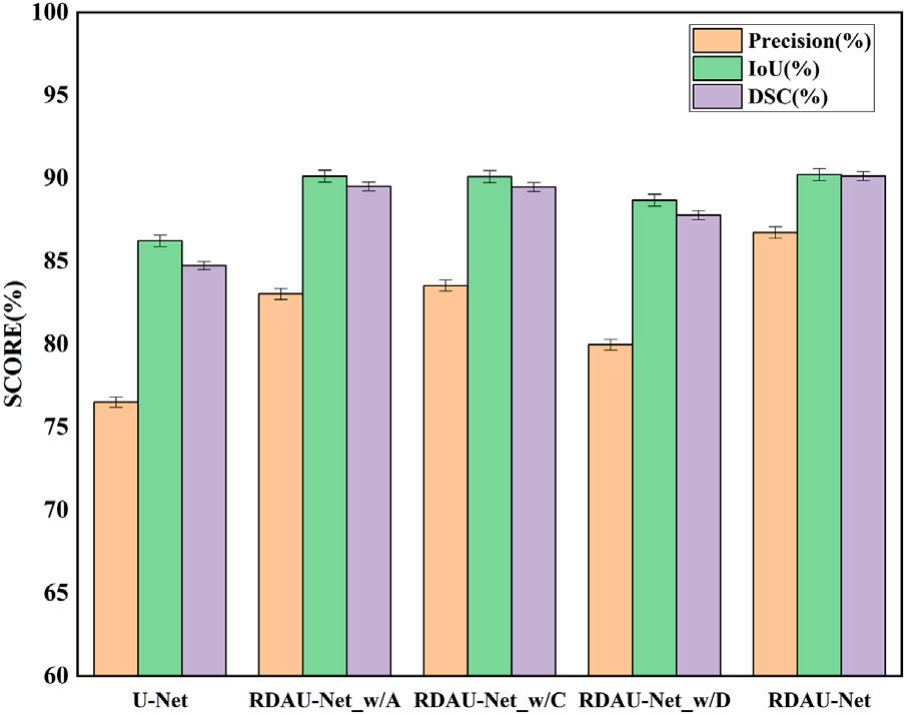
Comparison of segmentation indicators (*precision*, *IoU*, and *DSC*) of ablation models. Each of the added modules can improve the performance of the U-Net.

In the ablation experiments, the role of each module in the RDAU-Net model structure was studied, including the ablation attention mechanism, residual dynamic convolution module, and decoder multi-scale feature fusion. The three ablation models obtained are RDAU-Net_w/A of the ablation attention module, the RDAU-Net_w/D of the ablation residual dynamic convolution module, and the RDAU-Net_w/C of multi-scale feature fusion of the ablation decoder. The segmentation results and performance indicators of the ablation experimental model are shown in Figure 10 and Table 2, respectively. Compared with the basic model U-Net, the three ablation models have improved the performance of RDAU-Net_w/A and RDAU-Net_w/C which are closest to the segmentation performance of RDAU-Net. By comparison, it is found that the addition of the residual dynamic convolution module leads to the most obvious improvement in model performance, and compared with other modules, dynamic convolution can effectively reduce the probability of model misidentification. The RDAU-Net_w/D model is without the residual dynamic convolution modules, and its *precision* is 3.06% and 3.56% lower than RDAU-Net_w/A and RDAU-Net_w/C, respectively. The comparison results are shown in Figure 12.

**FIGURE 13:**
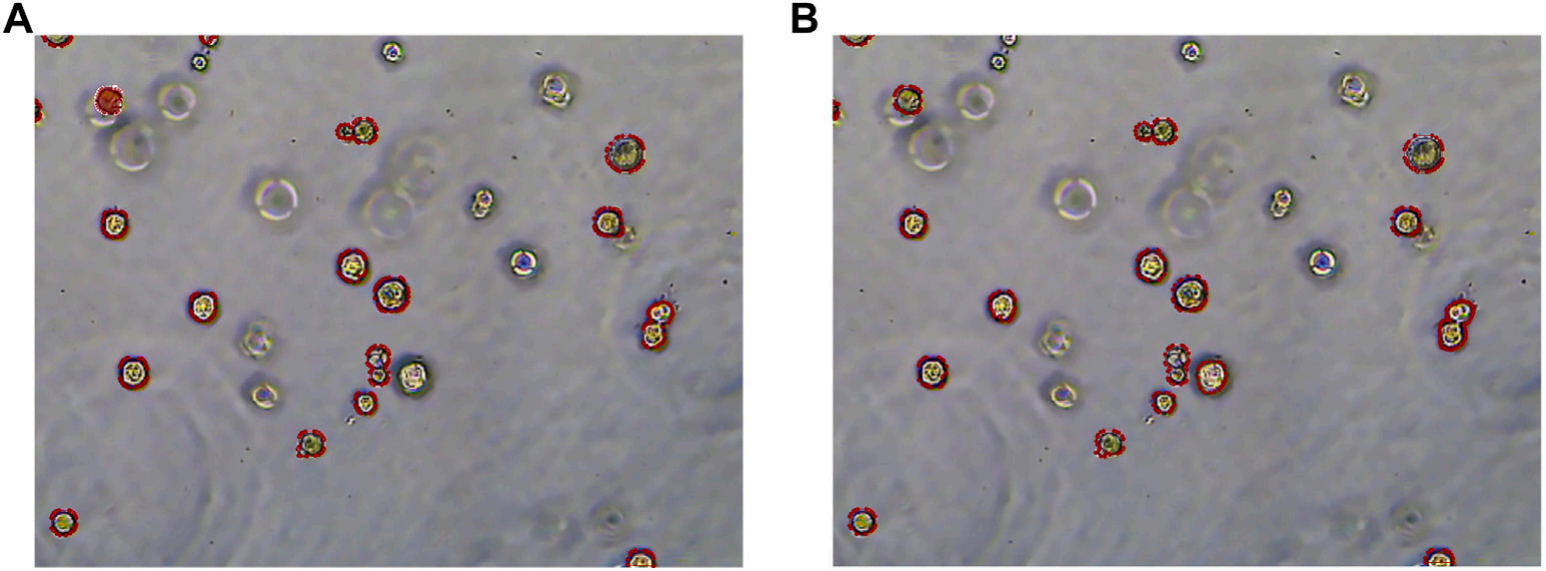
Segmentation results of the RDAU-Net model compared with manual annotation. **(A)** Manual labeling of organoids. **(B)** RDAU-Net model labeling of organoids (the red markers indicate targeted organoids). The comparison between Figure 13A and Figure 13B shows that the result of our model labeling is already very close to that of manual labeling.

To more clearly observe the model effect, the results of the expert label and the RDAU-Net model segmentation are restored to the original image for comparison. As shown in Figure 13, the segmentation result of the model is almost equivalent to the level of expert annotation, and the phenomenon of false identification is well controlled. As well, the improved RDAU-Net model can reduce the occurrence of false identification, and at the same time can reduce the phenomenon of missing identification and boundary defection of the target organoid region.

### 3.3 Drug screening evaluation

**FIGURE 14:**
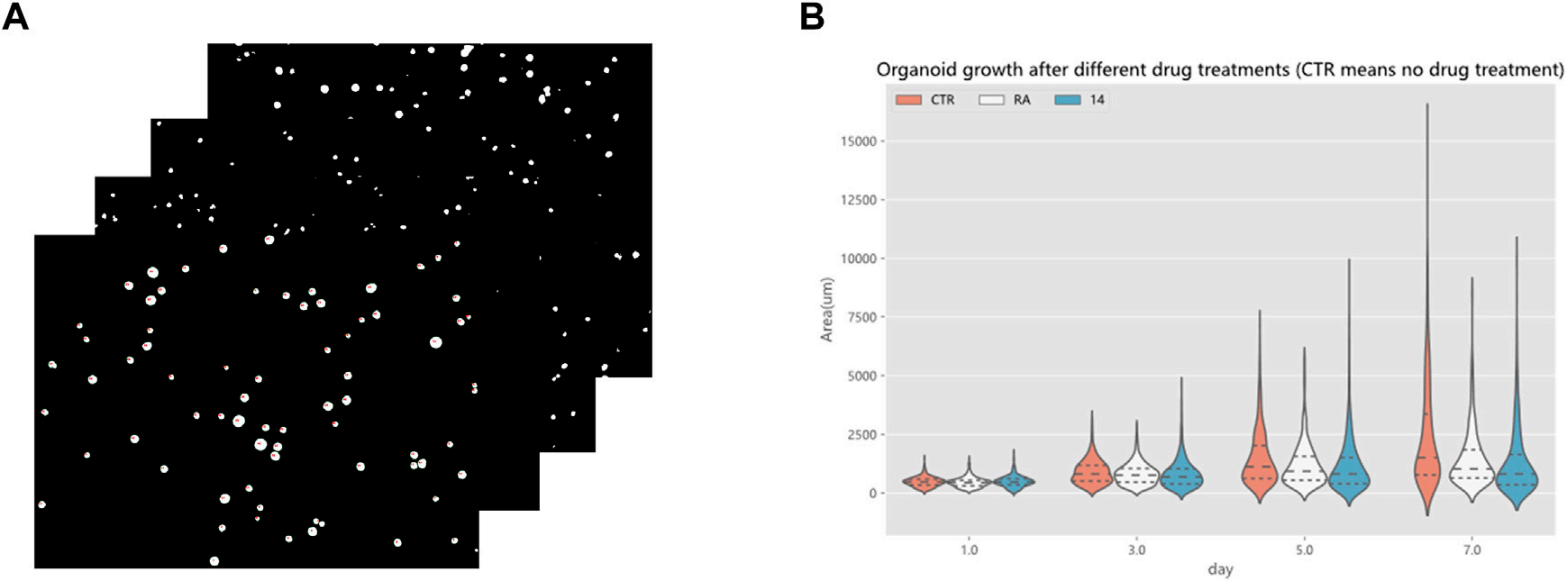
Drug screening evaluation by the RDAU-Net model. **(A)** Area statistics of the organoid in the images (only a small part is shown here). **(B)** Violin plot shows the area changes of organoids in CTR, RA, and 14 groups at days 1, 3, 5 and 7. The area data statistics of the organoids of different treatments on the same day can clearly show the difference between the size of the organoids (adding a quartile distribution map, the dotted line in the middle indicates the median of the data in this group).

Drug screening evaluation is one of the essential steps in drug development. It takes multiple experiments to show that screening drugs are effective in treating certain diseases (Tan et al., 2015), (Tan et al., 2017), (Tan et al., 2013). After the completion of the model training, a drug screening evaluation was performed. A *Python* program automatically calculated the area of organoids in the segmented organoid image and drew violin plots to document the growth status of organoids, which can then be used to determine the effectiveness of the drug. The violin plot in Figure 14 reflects the growth of the organoid in the three environments. The dataset used here for drug screening evaluation is not from the model training and testing dataset, but from additional data. Three sets of experiments are shown, CTR, RA, and 14. RA and 14 are two derivatives of vitamin A, and the CTR group is without drug treatment. Each set of experiments contained organoid images on days 1, 3, 5, and 7, with four or five organoid images per day, and 70 to 160 organoids per image. To make the comparison more intuitive and convenient, a quartile distribution map has been added to the violin plot, and the dotted line in the middle indicates the median of the data in this group. It can be concluded that 1–3 days is the initial growth stage of organoids, and there is no obvious growth difference between drug-treated organoids and non-drug-treated organoids. The differences gradually appear after the fifth day. The CTR group was not treated with drugs, and its organoid area peak and median were higher than those in the RA groups and the 14 groups. This suggests that these two drugs have a certain inhibitory effect on the growth of organoids from bladder cancer cell lines. We also analyzed the significance of differences in the distribution of organoids using the *t*-test, testing the significance of {CTR group and RA group} and {CTR group and 14 group} separately. There was no significant difference between the two groups on the first day, indicating that there was no significant difference in the area of plated organoids, which ensured the accuracy of the subsequent measurements. Only on the seventh day of culture, were there highly significant differences, showing that drug treatment can reduce the organoids area. To ensure the rigor of the experiment, the phenomenon reflected in Figure 14 must be confirmed by professionals in the relevant field to be correct and reliable. Therefore, by using the RDAU-Net model, we can automate the analysis of anti-cancer drug effects on organoids. By analogy, we can drive the development of anti-cancer drugs by screening tests for a broader variety of drugs in this way.

## 4 Discussion

The RDAU-Net model in this study is an improvement on the U-Net model to solve the shortcomings of the original model in organoid image segmentation. For example, the accuracy of organoid boundary segmentation is low, the boundary is not smooth enough, the anti-interference ability is poor, and the bubbles and organoids in the background are incorrectly identified. These issues have reduced the reliability of drug screening. The reasons for these problems are as follows: 1) the original double-layer convolutional feature extraction ability is not strong, and it is impossible to use multi-scale information for learning. 2) The original U-Net lacks attention modules, so it is easy to produce misidentification in the background with complex data, resulting in the reduction of segmentation accuracy. 3) There is a semantic gap between the high-level characteristics of the decoder and the low-level aspects of the encoder, due to which the recovery ability of the features is not strong. This study proposes corresponding improvement measures to overcome these problems. First, the original convolution is replaced by a dynamic convolution with residual connections. The organoids in the images are of different sizes and shapes, which makes segmentation difficult. The dynamic convolution automatically adjusts the parameters of the convolutional kernel based on the input, enhancing the adaptability of the model. It can achieve better segmentation results in the face of various organoids. Residual connections can accelerate model convergence and strengthen the stability of model training. Moreover, it can avoid vanishing gradients and gradient explosion. Adding different combinations of dilation rates to dynamic convolution can extract feature information of different scales by convolution, and fusing them significantly affects the segmentation of small-sized organoids. Second, in the face of the interference of background information, this study adds an attention mechanism to deal with it, which can not only strengthen the weight of target area features and suppress irrelevant areas but also capture long-distance information through the attention module, so that the model can make better use of the global information. From the comparison of U-Net and attention U-Net, RDAU-Net_w/A, and RDAU-Net in Table 2, it can be found that the *precision* indicator has been improved after adding the attention module. The higher the *precision*, the fewer false identifications the model has of organoids, and the stronger the model’s anti-interference ability to background information. Finally, the multi-scale feature fusion part of the decoder uses the multi-scale information to enhance the feature recovery ability of the decoder so that the model can perform better segmentation. It can be observed from Table 2 that compared with RDAU-Net_w/C, the recall indicator of RDAU-Net is improved. The *recall* indicator can reflect the miss recognition of the model. After observing the model, it is easy to miss the organoids with small areas and blurred boundaries. The multi-scale fusion of the decoder can effectively overcome this disadvantage.

The current research results can be used for drug screening and comparison in drug development. The use of computer-aided methods can significantly increase the efficiency of research development and reduce researchers’ workload. In contrast, this study only takes 15–20 min to segment 30 organoid images and draw a violin plot reflecting their growth patterns. It is much faster than the 4–6 h taken by manual screening and comparison. Although this study focused on bladder cancer cell line organoids, it could also be applied to drug screening and evaluation of other neoplastic cancer types.

## 5 Conclusion

In this study, we aim at the problem of drug screening using organoids derived from bladder cancer cell lines and propose a novel segmentation method based on deep learning. The proposed RDAU-Net model was developed based on the U-Net network. This novel model fills the semantic gap caused by jump connections and directly combines low-level and high-level features. In addition, the feature fusion of res-double Dy Conv modules and CA modules, as well as the decoder output portion, give the model the ability to extract multi-scale features and focus more on the target region. The application of artificial intelligence in anti-cancer drug screening here not only significantly reduces the time and economic cost of drug screening and study but also improves work efficiency and accuracy, suggesting a potential and broad prospect to apply this method in drug research and development. It was also found here that as the days passed, some of the organoids gradually aggregated together, making it difficult to distinguish them in a two-dimensional plane. In future, we plan to develop a 3D model to segment and reconstruct the organoids in 3D and calculate the volume of each organoid for the drug screening evaluation.

## Data Availability

The original contributions presented in the study are included in the article/Supplementary Material; further inquiries can be directed to the corresponding authors.
